# *In vitro* anti-proliferative activity of Argemone gracilenta and identification of some active components

**DOI:** 10.1186/s12906-015-0532-8

**Published:** 2015-02-05

**Authors:** Mario Alberto Leyva-Peralta, Ramón Enrique Robles-Zepeda, Adriana Garibay-Escobar, Eduardo Ruiz-Bustos, Laura Patricia Alvarez-Berber, Juan Carlos Gálvez-Ruiz

**Affiliations:** Departamento de Ciencias Químico Biológicas, Universidad de Sonora, Boulevard Luis Encinas y Rosales s/n, Hermosillo, Sonora 83000 Mexico; Centro de Investigaciones Químicas, Universidad Autónoma del Estado de Morelos, Av. Universidad No. 1001, Cuernavaca, Morelos 62209 Mexico

**Keywords:** Anti-proliferative activity, Cancer cells, *Argemone gracilenta*, Alkaloids, Fatty acids, MTT

## Abstract

**Background:**

Cancer is one of the leading causes of death worldwide. Natural products have been regarded as important sources of potential chemotherapeutic agents. In this study, we evaluated the anti-proliferative activity of *Argemone gracilenta’s* methanol extract and its fractions. We identified those compounds of the most active fractions that displayed anti-proliferative activity.

**Methods:**

The anti-proliferative activity on different cancerous cell lines (M12.C3F6, RAW 264.7, HeLa) was *evaluated in vitro* using the MTT colorimetric method. Identification of the active compounds present in the fractions with the highest activity was achieved by nuclear magnetic resonance (NMR) and gas chromatography-mass spectrometry (GC-MS) analyses.

**Results:**

Both argemonine and berberine alkaloids, isolated from the ethyl acetate fraction, displayed high anti-proliferative activity with IC_50_ values of 2.8, 2.5, 12.1, and 2.7, 2.4, 79.5 μg/mL on M12.C3F6, RAW 264.7, and HeLa cancerous cell lines, respectively. No activity was shown on the normal L-929 cell line. From the hexane fraction, a mixture of fatty acids and fatty acid esters of 16 or more carbon atoms with anti-proliferative activity was identified, showing a range of IC_50_ values of 16.8-24.9, 34.1-35.4, and 67.6-91.8 μg/mL on M12.C3F6, RAW 264.7, and HeLa cancerous cell lines, respectively. On the normal L-929 cell line, this mixture showed a range of IC_50_ values of 85.1 to 100 μg/mL.

**Conclusion:**

This is the first study that relates argemonine, berberine, and a mixture of fatty acids and fatty acid esters with the anti-proliferative activity displayed by *Argemone gracilenta*.

## Background

Cancer is one of the five leading causes of death, and by 2015 cancer morbidity is expected to climb to nine million people worldwide [1,2]. This growing trend indicates the deficiency in the current cancer therapies, which include surgery, radiotherapy, and chemotherapy [3,4]. There is a critical need for anti-cancer agents with higher efficacy, and less side effects that can be acquired at an affordable cost [2,5,6]. In this regard, plants represent a viable alternative because they have been valuable resources for traditional remedies since ancient times and continue to be the major source and inspiration for the development of therapeutic agents [7,8]. Some phytochemicals have been studied because of their inherent potential to cure diseases, as demonstrated by ancient medicinal practices [7,9]. Over 50% of anticancer drugs approved by the United States Food and Drug Administration since 1960 have been obtained from natural resources, especially from terrestrial plants [5,7]. Clinically important anticancer agents, such as paciltaxel, camptothecin, and vinblastine, and many other promising anticancer agents, currently under clinical trials, are also plant-derived compounds [1,10,11]. Mexico is considered a major supplier of natural resources. Within its great diversity of plants it is possible to find the *Argemone* genus*,* locally known as “cardo or chicalote” [12]. Species such as *Argemone mexicana*, *Argemone pleiacantha,* and *Argemone ochroleuca* have shown a variety of medicinal properties, such as antibiotic, sedative, analgesic, antimalarial, anti-inflammatory, and anti-tumor effects [12-16].

*Argemone gracilenta,* another species of the *Argemone* genus, grows in desert terrains mainly in the state of Arizona, South of the United States, and in the states of Sonora and Baja California Sur, northern Mexico. Previous studies have shown that *Argemone gracilenta* is relatively rich in alkaloids (0.33% of the dried plant), mainly (-)-argemonine that represents over 90% of the total plant alkaloids; other alkaloids have also been identified in smaller proportions such as (-)-mutagine, protopin, muramine, and (+)-reticuline [17].

Biological studies on *Argemone gracilenta* are scarce, and for this reason the aim of this work was to evaluate the anti-proliferative activity of this plant on different cancerous cell lines and to identify the responsible compounds for such activity.

## Methods

### General experimental procedures

Melting points were determined on a Fisher Johns melting point apparatus. The infrared spectra were measured on a Bruker Vector 22 spectrometer. GC-MS spectra were acquired using an Agilent 6890 series GC system and Agilent 5973 mass selective detector, employing a fused-silica column, 30 m × 0.32 mm HP-5MS (cross-linked 5% Ph Me silicone, 0.25 μm film thickness). The temperature of the column was varied from 40 to 250°C with a slope of 10°C/min and a stay of 5 min at this temperature. All NMR spectra were recorded on a Varian Unity 400 spectrometer at 400 MHz for ^1^H NMR, and 100 MHz for ^13^C NMR using DMSO-*d*_6_ and CDCl_3_ as solvents. Open column chromatographies were carried out on silica gel 60 (70–230 and 230–400 mesh [Merck]). Preparative TLC was performed on precoated silica gel 60 F254 plates (Merck).

### Plant material

*Argemone gracilenta* was collected in Guaymas, Sonora (28°05′57′ N, 111°03′23′ W), Northwest of Mexico, in May 2011. The plant was taxonomically identified (catalog No. 08274) by Jesús Sánchez Escalante, taxonomist at the Herbarium of the *Universidad de Sonora*.

### Extraction and fractionation

The plant was dried at room temperature and homogenized (1400 g) with a Whiley mill (200 mesh). An extract of the homogenized plant was obtained with methanol (1:10 w/v; plant/methanol) during 10 days under periodic agitation. The extract was filtered and concentrated to dryness on a rotatory evaporator under reduced pressure at 40°C. The methanol extract (130.9 g) was suspended consecutively in *n*-hexane, ethyl acetate, and ethanol (3 × 400 mL for each solvent) with constant agitation for 12 h. The volumes obtained (1200 mL) were concentrated to dryness under reduced pressure at 40°C to yield 26 g of *n*-hexane, 12.5 g of EtOAc, and 29.0 g of EtOH fractions. The methanol extract and fractions were stored to -4°C in amber glass vials until use [18].

The EtOAc fraction was further chromatographed on a silica gel column (120 g) eluting with *n*-hexane-CH_2_Cl_2_ (100:0 to 0:100), then CH_2_Cl_2_-MeOH (100:0 to 0:100) mixtures of increasing polarity to yield 11 fractions.

The FAg-4A fraction (9.7 mg, 0.24%), eluted with CH_2_Cl_2_-MeOH (98:2), was obtained as a yellow-orange oil.

The FAg-5B fraction (450 mg, 11.2%), eluted with CH_2_Cl_2_-MeOH (98:2), was obtained as a colorless oil.

The FAg 7 fraction (20. 5 mg, 0.51%), eluted with CH_2_Cl_2_-MeOH (85:15), was subjected to preparative TLC (CH_2_Cl_2_-MeOH, 8:2) to yield 6.3 mg of argemonine (Rf = 0.8) and 7.9 mg of berberine (Rf = 0.7).

Argemonine. Mp 122.3-123.4°C (melting point of reference 147-148°C [19]), ^**1**^H NMR (CDCl_3_, 400 MHz), δ ppm; 2.52 (d, J = 6.0 Hz, Hα-5 and Hα-11), 2.45 (s, N-CH_3_), 4.04 (dd, J = 6.61 Hz, H-6 and H-12) 3.58 (s, 3-OCH_3_ and 9-OCH_3_), 3.66 (2-OCH_3_ and 8-OCH_3_), 3.33 (d, J = 5.2 Hz, Hβ-5 and Hβ-11), 6.31 (s, H-1 and H-7), 6.47 (H-4 and H-10). ^13^C NMR (CDCl_3_, 100 MHz), δ ppm; 33.3 (C-5 and C-11), 39.7 (N-CH_3_), 56.3 (C-6 and C-12), 55.6 (3-OCH_3_ and 9-OCH_3_), 55.8 (2-OCH_3_ and 8-OCH_3_), 109.7 (C-4 and C-10), 111.3 (C-1 and C-7), 122.5 (C-4a and C-10a), 127.4 (C-1a and C-7a), 147.9 (C-3 and C-9), 148.4 (C21 and C-8).

Berberine. Mp 144.3-146.1°C (melting point of reference 146°C [19]) ^1^H NMR (CD_3_OD, 400 MHz), δ ppm; 3.26 (t, J = 5.6 Hz, H-5), 4.12 (s, 9-OCH_3_), 4.35 (s, 10-OCH_3_), 4.95 (t, J = 5.6 Hz, H-6), 6.13 (s, 3-OCH_2_O), 6.89 (s, H-4), 7.45 (s, H-1), 7.95 (d, J = 7.98, H-12), 8.00 (d, J = 7.98, H-11), 8.61 (s, H-13), 9.78 (s, H-8). ^13^C NMR (CD_3_OD, 100 MHz), δ ppm; 28.2 (C-5), 57.1 (C-6), 57.6 (9-OCH_3_), 62.5 (10-OCH_3_), 103.6 (2,3-OCH_2_O), 106.5 (C-1), 109.3 (C-4), 121.5 (C-13), 121.9 (C-4a), 123.3 (C-12a), 124.5 (C-12), 128 (C-11), 131.9 (C-14a), 135.1 (C-8a), 139.6 (C-14), 145.7 (C-9), 146.4 (C-8), 149.9 (C-3), 152 (C-2 and C-10).

### GC-MS analysis of fractions Fag-4A and Fag-5B

The content of fatty acids and fatty acid esters in fractions FAg-4A and FAg-5B was analyzed by gas chromatography (Agilent 6890) coupled to a quadrupole mass detector in electron impact mode at 70 eV (Agilent 5973 N). A solution of 5 mg of each subfraction in 1 mL of solvent (methanol or dichloromethane) was prepared. Volatile compounds were separated on an HP 5MS capillary column (25 m long, 0.2 mm i.d., 0.3 μm film thickness). The oven temperature was set at 40°C for 2 min and then programmed from 40 to 260°C at 10°C/min, and kept for 20 min at 260°C. Mass detector conditions were as follows: interphase temperature was 200°C and mass acquisition ranged from 20 to 550. Temperatures of the injector and detector were set to 250°C and 280°C, respectively. The splitless injection mode was performed with 1 μL of the oily extract. The carrier gas was helium at a flow rate of 1 mL/min. Volatiles were identified by comparing their mass spectra with those of the National Institute of Standards and Technology NIST 1.7 library. Semi-quantitative data were calculated from the GC peak areas without using correction factors and were expressed as relative percentage (peak area %) of the total volatile constituents identified.

### Cell lines and cell culture

The M12.C3F6 (murine B-cell lymphoma) and RAW 264.7 (macrophage, transformed by Abelson murine leukemia virus) cells lines were kindly provided by Dr. Emil R. Unanue (Department of Pathology and Immunology, Washington University in St. Louis, MO). Cell lines NCTC clone L-929 (normal subcutaneous connective tissue) and HeLa (human cervix carcinoma) were purchased from the American Type Culture Collection (ATCC, Rockville, MD). All cell cultures were carried out in Dulbecco’s modified Eagle’s medium (DMEM) supplemented with 5% heat- inactivated fetal calf serum and grown at 37°C in an atmosphere of 5% CO_2_.

### Cell viability assay

The MTT assay was used to evaluate the anti-proliferative activity. It is a colorimetric assay based in the fact that mitochondrial oxidoreductase enzymes are capable of reducing the tetrazolium dye MTT 3-(4,5-dimethylthiazol-2-yl)-2,5-diphenyltetrazolium bromide to its insoluble formazan, which has a purple color. The cellular oxidoreductase enzymes may, under defined conditions, reflect the number of viable cells present. Briefly, cells were seeded in a 96-well plate with DMEM medium (high glucose, supplemented with 5% FBS) at a density of 10,000 cells/well. Different concentrations of methanol extract and fractions were added followed by 48 h incubation. All experiments were conducted in parallel with controls (0.06%-0.5% DMSO). Ten microliters of 3-(4,5-dimethylthiazol-2-yl)-2,5-diphenyltetrazolium bromide (MTT, 5 mg/mL; Sigma, USA) were added to each well at the end of the treatment period and incubated at 37°C for 4 h. Formazan crystals were dissolved with acidic isopropanol, and the plates were read in an ELISA plate reader, using a test wavelength of 570 nm and a reference wavelength of 630 nm. Plates were normally read within 10 min after adding isopropanol. The anti-proliferative activity of extracts was reported as IC_50_ values (IC_50_ value was defined as the concentration of extract that inhibits cell proliferation by 50%) [20].

### Statistical analysis

All data were expressed as mean ± SD. Data were subjected to statistical analysis of variance (ANOVA) by comparing means with Tukey test (p <0.05). IBM® SPSS® 20 statistical program was used for all statistical analyses.

## Results and discussion

Anti-proliferative activity assays were performed using the MTT colorimetric assay on three cancerous cell lines (M12.C3F6, RAW 264.7, and HeLa) and a normal cell line (L-929) as control. The concentrations used of the methanol extract and fractions were from 25 to 200 μg/mL, each assay was performed in triplicate.

Table 1 shows the results of the *in vitro* anti-proliferative activity assays of the *Argemone gracilenta’s* methanol extract and its fractions on different cancerous cell lines. The results are expressed in IC_50_ values (mg/mL) ± SD. The United States National Cancer Institute (US-NCI) establishes that a crude extract that shows an IC_50_ value of less than 100 μg/mL is considered active. When the IC_50_ value is lower than 30 μg/mL, the US-NCI considers a crude extract promising for purification and a biological activity study [3,21]. In the case of pure compounds, they are considered active when the IC_50_ values are lower than 4 μg/mL [22,23].

**Table 1:** **Anti-proliferative activity (IC**_**50**_ **values) of the methanol extract and fractions of** ***Argemone gracilenta*** **on selected cancer cell lines**

**Cancer cell line**	**IC**_**50**_ **(μg/mL)**				
	**Methanol**	**Hexane**	**Ethyl acetate**	**Ethanol**	**Residual**
M12.C3F6	46.20 ± 8.41ª	20.40 ± 2.30^c^	32.60 ± 1.10^b^	21.08 ± 0.84^c^	40.60 ± 10.08^a^
RAW 264.7	64.45 ± 8.97ª	36.06 ± 6.55^d^	41.27 ± 4.27^c^	55.18 ± 8.80^b^	64.65 ± 5.36ª
HeLa	78.87 ± 8.52^b^	70.62 ± 5.80^c^	126.28 ± 5.73ª	> 200*	> 200*
L-929	160.60 ± 2.15^b^	131.30 ± 3.39^c^	180.61 ± 4.37ª	> 200*	> 200*

Data are shown as the mean ± SD from three independent repeats after a 48-h exposure to the test extract and fractions. Significant differences (p < 0.05) are indicated by different letters (a-d). The asterisk (*) represents the maximum concentration tested that did not reach IC_50_ values.

The methanol extract showed activity on the M12.C3F6 cell line with an IC_50_ value of 46.20 ± 8.41 μg/mL; for RAW 264.7 and HeLa cell lines, the extract showed activity with IC_50_ values of 64.45 ± 8.97 and 78.87 ± 8.52 μg/mL, respectively. The methanol extract was not active on the normal cell line L-929 used as control, since it showed an IC_50_ value > 100 μg/mL.

After fractionation of the methanol extract, there was an increment in the activity of some fractions. The ethyl acetate fraction had greater activity, with an increase in the anti-proliferative activity mainly on cell lines RAW 264.7 and M12.C3F6 with IC_50_ values of 32.60 ± 1.10 and 41.27 ± 4.27 μg/mL respectively, and showing no activity on the normal cell line L-929, with an IC_50_ value > 100 μg/mL.

Chromatographic separation of the ethyl acetate fraction yielded 11 subfractions; of these, fractions Fag-4A, Fag-5B, and the pure compounds argemonine (7A) and berberine (7B) showed anti-proliferative activity on M12.C3F6, RAW 264.7, and HeLa cancer cell lines, and on the L929 control cell line (Table 2). The alkaloid argemonine was the most active, with IC_50_ values of 2.8 and 2.5 μg/mL on M12.C3F6 and RAW 264.7 cell lines, respectively (Table 2). Comparing these results with the methanol extract activity indicates that the activity increased, since argemonine was 25-times more active than the methanol extract on the RAW 264.7 cell line. In the HeLa cell line, argemonine showed activity with an IC_50_ value of 12.1 μg/mL, which is 6-times more active than the methanol extract. On the normal cell line L-929, it was not active, indicating that argemonine is selective for cancer cell lines.

**Table 2:** **Anti-proliferative activity (IC**_**50**_ **values) of the most active subfractions from the ethyl acetate fraction on selected cancer cell lines**

**Cancer cell line**	**IC**_**50**_ **(μg/mL)**			
	**FAg-4A**	**FAg-5B**	**FAg-7A**	**FAg-7B**
M12.C3F6	16.81 ± 4.0^b^	24.9 ± 2.2^a^	2.8 ± 0.3^c^	2.4 ± 0.4^c^
RAW 264.7	34.1 ± 5.8^b^	35.4 ± 10.14^b^	2.5 ± 0.5^b^	2.7 ± 0.1^b^
HeLa	91.8 ± 7.6^a^	67.61 ± 5.6^c^	12.1 ± 1.7^d^	79.5 ± 11.5^b^
L-929	151.7 ± 1.5^a^	85.1 ± 2.3^b^	> 100*	> 100*

Data are shown as the mean ± SD from three independent repeats after a 48-h exposure to the test subfractions. Significant differences (p < 0.05) are indicated by different letters (a-d). The asterisk (*) represents the maximum concentration tested that did not reach IC_50_ values.

Morphological changes in cells caused by the effect of a compound or fraction isolated from a plant can provide information about the cell death mechanism activated in such cells. Various cell death pathways, including apoptosis, autophagy, oncosis, etc., have been proposed. Each of them is characterized by certain morphological changes that can be used to distinguish them through observation under the microscope. Apoptosis is characterized by a nuclear and cytoplasm condensation and cellular fragmentation into membrane-bound fragments (apoptotic bodies). In oncosis, the cell demonstrates swelling, rapid membrane breakdown, swollen nuclei without DNA fragmentation, and organelle swelling. In autophagy, vacuolization (autophagic vacuoles), degradation of cytoplasm contents, and a slight chromatin condensation are observed [24].

**Figure 1:**
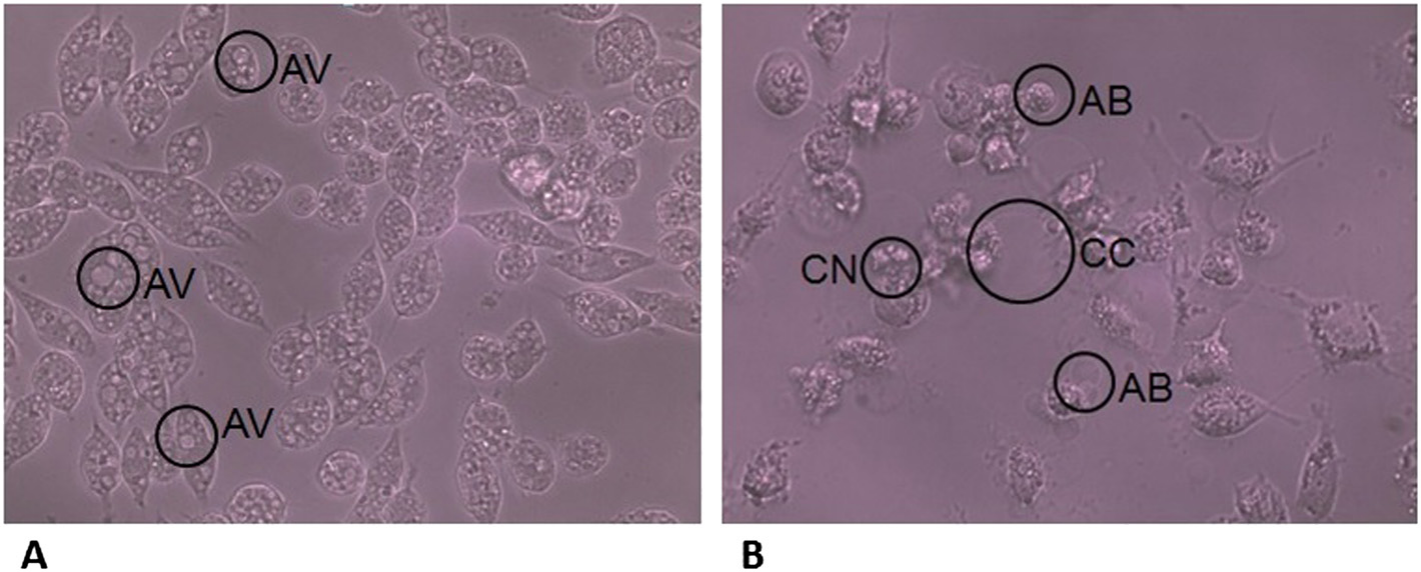
**Anti-proliferative activity of the Fag-7A (argemonine) fraction on cell lines. A)** M12.C3F6 and **B)** HeLa, at a concentration of 50 μg/mL. Observation at 48 h in inverted microscope. Morphological changes: condensed nucleus (CN), apoptotic bodies (AB), autophagic vacuoles (AV), condensed cytoplasm (CC). All images are magnified at 40×. Images shown are representative of at least four such fields of view per sample and three independent trials.

The cell lines were observed 48 h after exposure to argemonine and evidence of two different cell death pathways was found. In the M12.C3F6 cell line (Figure 1A), autophagic vacuoles and degradation of cytoplasm contents, characteristic morphological changes of cell death by autophagy, were observed. In the HeLa cell line (Figure 1B), condensation of the nucleus and cytoplasm, and apoptotic body formation, characteristic morphological changes of cell death by apoptosis, were present.

### Structural analysis of the active compounds

The composition of FAg-4A and FAg-5B fractions were obtained by GC-MS analysis (Table 3). The two fractions are composed of fatty acids of 16 or more carbon atoms. The FAg-4A fraction is a mixture of: 7-octadecanoic acid, methyl ester (40.83%), hexadecanoic acid, methyl ester (33.39%), hexadecanoic acid, ethyl ester (14.98%), and linoleic acid, ethyl ester (10.78%). The FAg-5B fraction consists of: 9,12-octadecadienoic acid (81.63%), hexadecanoic acid (9.05%), 7-octadecenoic acid, methyl ester (8.39%), and hexadecanoic acid, methyl ester (1.01%).

**Table 3:** **Composition of Fag-4A and Fag-5B fractions (GC-MS)**

**Fraction**	**Compound**	**Retention time (min)**	**Percentage (%)**
FAg-4A	Hexadecanoic acid, methyl ester	18.62	33.39
	Hexadecanoic acid, ethyl ester	19.28	14.98
	7-Octadecenoic acid, methyl ester	20.34	40.83
	Linoleic acid ethyl ester	20.95	10.78
FAg-5B	Hexadecanoic acid, methyl ester	18.62	1.01
	Hexadecanoic acid	19.3	9.05
	7-Octadecenoic acid, methyl ester	20.31	8.39
	9,12-Octadecadienoic acid	21.11	81.63

**Figure 2:**
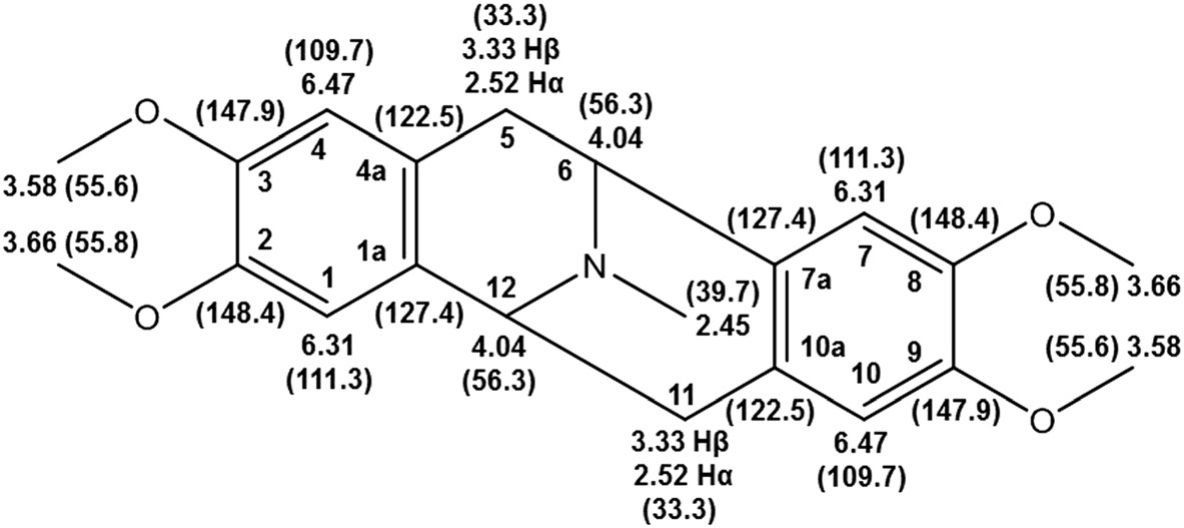
**Structure of the main component in Fag-7A fraction, argemonine:** ^**1**^ **H NMR (** ^**13**^ **C NMR) data.**

**Figure 3:**
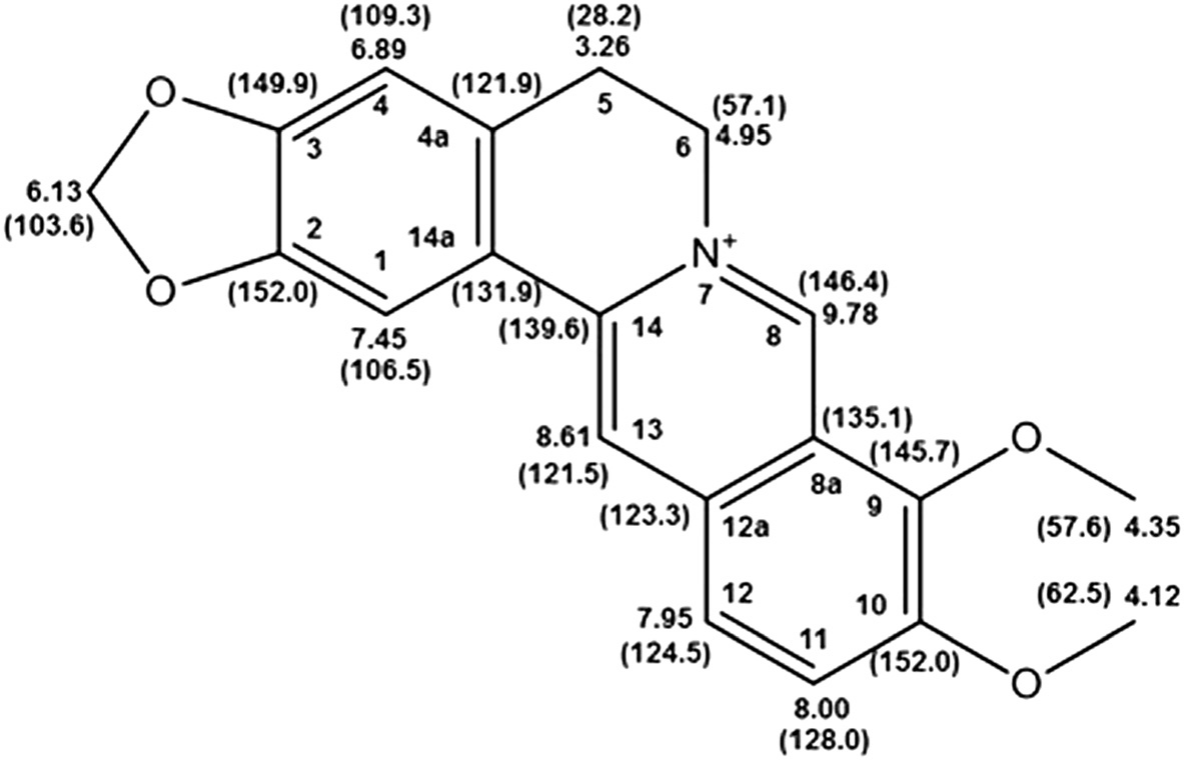
**Structure of the main component in Fag-7B fraction, berberine:** ^**1**^ **H NMR (** ^**13**^ **C NMR) data.**

The compounds argemonine (7A [25] and berberine (7B [26]) were identified by comparing their spectroscopic data with those previously described in the literature (Figures 2 and 3). The purity of isolated compounds was determined to be above 95%, based on their ^1^H-NMR spectra.

Argemonine is a natural alkaloid from the isoquinoline group, which has been isolated from plants and seeds of several species, including some plants of the genus *Argemone*, such as *A. gracilenta*, *A. platyceras*, *A. sanguinea*, among others. In addition, it has been found also in species of *Buxifolia berberis*, *Thalictrum revolutum,* and *T. strictum*. The most popular use of argemonine is to control pests in crops, in combination with berberine and ricin, because of its antibacterial, fungicidal, and insecticidal properties [27]. Biological studies of argemonine are scarce, and its biological activities have not been determined yet.

Previous studies have shown that other types of isoquinoline alkaloids, such as sanguinarine and chelerythrine, isolated also from species of the *Argemone* genus, showed anti-proliferative activity on several cancer cell lines such as HeLa, MCF-7, A-549, and PC-3 [28]. It has been pointed out that sanguinarine induces cell cycle arrest in different phases and apoptosis in a variety of cancer cells [29], besides possessing a wide spectrum of biological activities, such as antimicrobial, antifungal, and anti-inflammatory effects.

Studies on berberine have shown its ability to inhibit the growth of various human cancer cell lines. These studies have proven that berberine suppresses cancer cell proliferation by regulating the cell cycle [3,30-32]. In 2011, it was reported that berberine induces cell death by autophagy in hepatocellular carcinoma cell lines HepG2 and MHCC97. Berberine exerts an inhibitory effect on invasion, migration, metastasis, and angiogenesis of cancer cells [33,34]. These results illustrate the potential application of berberine in cancer therapy [3].

Anti-proliferative activity studies have shown that certain fatty acid compounds inhibit the growth of cancer cells. Girao evaluated the effect of 18-carbon fatty acids on the SP210 cell line (mouse myeloma) growth, demonstrating that unsaturated 18 carbon fatty acids exert anti-proliferative activity on that cell line, whereas saturated fatty acids (C18.0, stearic acid) show no cell inhibition activity [35]. However, other studies have demonstrated that some fatty acids stimulate the growth of cancer cells. For example, arachidonic acid (C-20: 4) stimulates the growth of human prostate cancer cell line, PC-3, by 122%, but these studies also found that fatty acids, such as omega-3 eicosapentaenoic acid, exert an inhibitory effect on the growth of PC-3 cells [36].

Studies of biological activities and the importance of fatty acids extracted from the *Argemone* genus are scarce. For this reason, the results regarding the anti-proliferative activity of fatty acids, FAG-4A and FAG-5B fractions, could be the starting point for their further study as potential inhibitors of a wide range of human cancer cells, hence, pointing out their relevance in the battle against cancer.

## Conclusions

This study presents the first analysis of the anti-proliferative activity of *Argemone gracilenta* on cancerous cell lines and provides support for the traditional use of this plant against multiple diseases, as well as of other species of the *Argemone* genus. In addition, two alkaloids, berberine and argemonine, with important anti-poliferative activity were isolated. We present the first analysis of the argemonine alkaloid as an anti-proliferative compound, showing promising results for future studies as a potential anticancer drug. Fatty acids and fatty acids esters of 16 or more carbon atoms with anti-proliferative activity were also identified. This is also the first time that the anti-proliferative activity displayed by plants of the *Argemone* genus is associated to this type of compounds.
